# Supplementary material for: Does the use of statins alter the risk of rheumatoid arthritis? A systematic review and meta-analysis
Source: PLoS One. 2024 Jul 23;19(7):e0307599. doi: 10.1371/journal.pone.0307599 (PMC11265694; doi:10.1371/journal.pone.0307599)
Supplement: Attachment — Submitted filename: COMMENTS.docx [file pone.0307599.s002.docx]

Journal Requirements:

When submitting your revision, we need you to address these additional requirements.

1. Please ensure that your manuscript meets PLOS ONE's style requirements, including those for file naming. The PLOS ONE style templates can be found at 
https://journals.plos.org/plosone/s/file?id=wjVg/PLOSOne_formatting_sample_main_body.pdf and 
<https://journals.plos.org/plosone/s/file?id=ba62/PLOSOne_formatting_sample_title_authors_affiliations.pdf>

**Response**: Done

2. We note that your Data Availability Statement is currently as follows: [All relevant data are within the manuscript and its Supporting Information files.]

Please confirm at this time whether or not your submission contains all raw data required to replicate the results of your study. Authors must share the “minimal data set” for their submission. PLOS defines the minimal data set to consist of the data required to replicate all study findings reported in the article, as well as related metadata and methods (https://journals.plos.org/plosone/s/data-availability#loc-minimal-data-set-definition).

For example, authors should submit the following data:

- The values behind the means, standard deviations and other measures reported;
- The values used to build graphs;
- The points extracted from images for analysis.

Authors do not need to submit their entire data set if only a portion of the data was used in the reported study.

If your submission does not contain these data, please either upload them as Supporting Information files or deposit them to a stable, public repository and provide us with the relevant URLs, DOIs, or accession numbers. For a list of recommended repositories, please see https://journals.plos.org/plosone/s/recommended-repositories.

If there are ethical or legal restrictions on sharing a de-identified data set, please explain them in detail (e.g., data contain potentially sensitive information, data are owned by a third-party organization, etc.) and who has imposed them (e.g., an ethics committee). Please also provide contact information for a data access committee, ethics committee, or other institutional body to which data requests may be sent. If data are owned by a third party, please indicate how others may request data access.

**Response**: The article contains all data analyzed in the meta-analysis

3. PLOS requires an ORCID iD for the corresponding author in Editorial Manager on papers submitted after December 6th, 2016. Please ensure that you have an ORCID iD and that it is validated in Editorial Manager. To do this, go to ‘Update my Information’ (in the upper left-hand corner of the main menu), and click on the Fetch/Validate link next to the ORCID field. This will take you to the ORCID site and allow you to create a new iD or authenticate a pre-existing iD in Editorial Manager. Please see the following video for instructions on linking an ORCID iD to your Editorial Manager account: <https://www.youtube.com/watch?v=_xcclfuvtxQ>

**Response**: Done

4. We note that you have referenced (unpublished) on page 4,  which has currently not yet been accepted for publication. Please remove this from your References and amend this to state in the body of your manuscript: (ie “Bewick et al. [Unpublished]”) as detailed online in our guide for authors
http://journals.plos.org/plosone/s/submission-guidelines#loc-reference-style

**Response**: We have written on page 4 that: “The reviewers excluded cross-sectional studies, editorials, and unpublished data.” This means we have not included any unpublished data in the manuscript.

**Comments to the Author**

Reviewer #1: This is a systematic review and meta-analysis on the impact of statins on the risk of rheumatoid arthritis. Although simple, was very well conducted and discussed considering scientific knowledge. I congratulate the authors for their manuscript.

**Response**: Thank you for your comments.

Reviewer #2: The authors conducted a systematic review and meta-analysis evaluating the potential link between statin use and the risk of developing rheumatoid arthritis. However, as mentioned by the authors, this topic had been clearly addressed by the previous published meta-analysis (doi: 10.1016/j.semarthrit.2020.03.008). There are 2 studies (Tascilar [2016] and Chodick [2010]) had been included in the previous one but not the current submission. Please clarify the reasons for not including those two studies. In addition, there are some issues regarding the methodology used in this submission.

**Response**: Thank you for reviewing our manuscript and providing constructive comments. We agree with the reviewer that 2 studies (Tascilar [2016] and Chodick [2010]) included in the previous review were not included in our review, but for important reasons. The study of Tascilar 2016 is in complete overlap with de Jong 2018. Since de Jong 2018 had a longer study duration and larger sample size, we excluded Tascilar 2016. The study of Chodick et al assessed the risk of bias with “persistence of statins”. In their cohort all patients were users of statins and they divided their study population into multiple groups based on number of days of covered with statins (<20%, 20-29%,…>80%). There was no control group of “no statins” in their study. Hence, this study was not included. We have edited Figure 1 to update the reasons for exclusion as these were not reflected earlier.

1. The authors included both randomized and non-randomized studies, which is not recommended by the Cochrane Handbook due to the risk of bias.

**Response**: We agree with the point of view of the reviewer. Hence, we have already conducted a subgroup analysis based on study designs. Our aim was to present comprehensive evidence on the association between statin use and risk of RA. This would have been incomplete without presenting the results of the only RCT conducted on this topic.

2. The authors assessed the quality of all included studies by using the Newcastle Ottawa Scale (NOS), but it is a tool used only for the non-randomized studies. For randomized controlled trials, the Cochrane Risk of Bias (RoB) is recommended.

**Response**: This error is now corrected. We have now used the Cochrane Risk of Bias tool. Since there was only one RCT, results are presented in the manuscript itself with the following lines: “The RCT was found to have low risk of bias for randomization process, deviation from intended intervention, measurement of outcomes, and selection of reported results. However, there was high risk of bias due to missing outcome data owing to high number of drop-outs. Hence, overall the risk of bias for the trial was high.”

3. The authors mentioned that “Given the small risk of RA with the exposure to statin, odds ratios, hazard ratios, and risk ratios were considered to have minimal differences and were combined in a single meta-analysis.”, which I think is incorrect. I have checked the included studies and found even both odds ratios (ORs) reported in the articles (for example, Schmidt [2013] and de Jong [2012]), there were significant differences in the way they calculated the ORs. This could also be one of reasons for the high heterogeneity. I would suggest using raw data (number of events) and pooled with the same ratio (OR or RR).

**Response**: We acknowledge this limitation of our review since some of the studies used OR while some used HR to report the association between statins and RA. This is now incorporated in the limitation section of the review. “Another factor to consider is that the included studies used different ratios, OR or hazard ratio or risk ratio to report the association between statin use and RA. Due to low prevalence of RA and limited number of studies in literature, we were forced to combine these ratios in a single meta-analysis. Given these ratios are not exactly the same, there could be some bias in our results.”

Since the number of studies in this review is limited and ratios differ, we are unable to separate studies based on the ratios used. Further, we stand by the statement made in the manuscript that in cases of low outcome rates (in this case RA), the different ratios are combined together in meta-analysis studies. This was also done in the prior systematic review of Myasoedova et al, quoted above by the reviewer, wherein they stated that: “The primary effect measures used in the studies were Odds Ratios (OR), Hazard Ratios (HR) and Relative Risks (RR). These effect measures were assumed to reasonably estimate the same association between statin use and RA occurrence given the low incidence of RA and thus were pooled together”

Secondly, we would like to stick with a meta-analysis of adjusted ratios as used in our meta-analysis and also the prior meta-analysis and not go in for raw data meta-analysis. Crude/raw data analysis is not adjusted for confounders and hence more prone to bias than combining different ratios together.

4. As several of the included studies mentioned that intervention durations of less than or more than 1 year may affect the results. Subgroup analysis based on intervention duration is needed.

**Response**: As shown in Table 2, where we have collated all subgroup analysis reported by the included studies, only three studies reported data on the effect of duration of statin use and risk of RA. Peterson et al and de Jong 2018 classified as > or <1 year while de Jong 2012 classified the duration as 1-250 days, 251–600 days and ≥601 days. Given the scarce data and variation in classification, it is not possible to pool them into a subgroup analysis. Secondly, none of the remaining studies reported data on duration of consumption of statins to allow a subgroup analysis on this important variable. Lastly, follow-up data was also inconsistently reported by the studies which further doesn’t allow a subgroup analysis based on duration of follow-up. All these are already acknowledged in the limitation section of the discussion.

5. It is also important to conduct the leave-one-out analysis to determine whether the results could have been influenced by a single study.

**Response**: Sensitivity analysis is now added to the manuscript.

*.*
